# Classifying visuomotor workload in a driving simulator using subject specific spatial brain patterns

**DOI:** 10.3389/fnins.2013.00149

**Published:** 2013-08-21

**Authors:** Chris Dijksterhuis, Dick de Waard, Karel A. Brookhuis, Ben L. J. M. Mulder, Ritske de Jong

**Affiliations:** ^1^Department of Psychology, University of GroningenGroningen, Netherlands; ^2^Department of Infrastructure Systems and Services, Delft University of TechnologyDelft, Netherlands

**Keywords:** passive brain computer interface, common spatial pattern, driving simulator, workload classification, adaptive automation, lateral control

## Abstract

A passive Brain Computer Interface (BCI) is a system that responds to the spontaneously produced brain activity of its user and could be used to develop interactive task support. A human-machine system that could benefit from brain-based task support is the driver-car interaction system. To investigate the feasibility of such a system to detect changes in visuomotor workload, 34 drivers were exposed to several levels of driving demand in a driving simulator. Driving demand was manipulated by varying driving speed and by asking the drivers to comply to individually set lane keeping performance targets. Differences in the individual driver's workload levels were classified by applying the Common Spatial Pattern (CSP) and Fisher's linear discriminant analysis to frequency filtered electroencephalogram (EEG) data during an off line classification study. Several frequency ranges, EEG cap configurations, and condition pairs were explored. It was found that classifications were most accurate when based on high frequencies, larger electrode sets, and the frontal electrodes. Depending on these factors, classification accuracies across participants reached about 95% on average. The association between high accuracies and high frequencies suggests that part of the underlying information did not originate directly from neuronal activity. Nonetheless, average classification accuracies up to 75–80% were obtained from the lower EEG ranges that are likely to reflect neuronal activity. For a system designer, this implies that a passive BCI system may use several frequency ranges for workload classifications.

## Introduction

In contrast to an active Brain-Computer Interface (BCI) which allows users to engage in volitional thought control of a device, several BCI researchers have proposed to advance human-computer interaction by triggering machine actions based on inferences of the user's current mental state, known as passive BCI (Kohlmorgen et al., 2007; Cutrell and Tan, 2008; Zander et al., 2010; Zander and Kothe, 2011). For example, Kohlmorgen et al. (2007) showed that it is possible to classify mental workload elicited by a secondary task mimicking cognitive processes in a real driving environment. Moreover, these classifications were then used to switch on and off a tertiary task that mimicked an interaction with the vehicle's electrical devices that in turn improved performance on the secondary task.

In the human factors and ergonomics literature, which traditionally focuses on overall system performance and safety critical tasks, the potentially detrimental effects of both mental underload and overload have been a major research topic for decades. Mental workload can be defined as a “reaction to demand” and “the proportion of capacity that is allocated for task performance” (de Waard, 1996). Mental underload and overload both represent compromised functional states during which a breakdown of primary task performance is more likely (e.g., Hockey, 1997, 2003; see also Brookhuis and de Waard, 2010). Preventing these hazardous functional states by maintaining mental workload or task demand within an acceptable range in real-time has been the central goal of adaptive automation since the seventies (Chu and Rouse, 1979; Hancock and Chignell, 1988; Rouse, 1988; Parasuraman et al., 1992; Kaber and Prinzel, 2006).

A large part of adaptive automation literature is devoted to determining the right moment of providing or withdrawing task support, and several types of triggers may be available to optimize performance of a human-machine system (e.g., critical events and human task performance; see Parasuraman et al., 1992). Therefore, the question arises as to what physiological measures could offer in terms of improving the overall system's performance. The most important argument for the inclusion of physiological measures in a control loop is their potential for detecting user states that would otherwise remain hidden. Human beings may exhaust themselves to protect primary task performance in demanding situations. While performance protection is important for dealing with short bursts of task demand, when exposed to longer periods of high workload, it may have affective costs such as increases in anxiety, but also compensatory performance costs, such as neglecting secondary tasks (Hockey, 1997, 2003). Since straining effort expenditure has a neurophysiological base, the ability to reliably classify workload using physiological measures could be used to offload a person, before performance effects become apparent.

Traditional research approaches might not be well suited for uncovering the underlying neurophysiological mechanisms that could be used in a support system. As pointed out by Fairclough (2009), the fundamental problem of using physiological measures is the complex relationship between user states, such as mental overload, and their associated physiological variables. Specifically, four physiology-to-state mappings can be distinguished (Cacioppo et al., 2000). In the most straightforward case, there is a unique *one-to-one* mapping between a physiological variable and the psychological construct. Such a unique, one-to-one mapping would be ideal for an interactive system. However, a one-to-one mapping that holds true in both the laboratory and the field has to date not yet been found. A *many-to-one* mapping is more complicated as several signals are needed to infer a mental state. For example, heart rate, heart rate variability and blood pressure have been combined to infer mental workload (e.g., Mulder et al., 2009). In a *one-to-many* mapping, one physiological signal responds to a range of user states. For instance, systolic blood pressure information was found to be sensitive to excitement, frustration, and stress (Cacioppo and Gardner, 1999). Lastly, the most common finding is a *many-to-many* mapping where many signals are in fact sensitive to many mental states. Ultimately, in an implicit human-machine control loop, a one-to-one or a many-to-one relation is required. As briefly mentioned, another factor complicating the relationship between physiological measures and user state is lack of generalizability outside the laboratory setting where a mapping was found. Simply put, a relation between a physiological measure and a user state found in the laboratory may not hold true in a real world setting where environmental conditions are less controlled.

Furthermore, due to large individual differences in physiological responsiveness, traditional statistical tests might not be suitable to uncover relationships that are valuable for implicit machine control. Even in a repeated measures analysis of variance, where the variations due to individual differences are partly taken out of the error term, the directions of effects within the individuals need some consistency across individuals to reach statistical significance. While significant effects on a group level are interesting from a fundamental point of view, individual patterns are more relevant, when physiology is applied in human-machine systems. In this respect, the feature extraction and classification algorithms used by BCI researchers offer a promising way of dealing with these limitations.

As shown by Kohlmorgen et al. (2007), driver support may be linked to electroencephalogram (EEG) signals. Given the fact that the driving task is increasingly demanding, due to increased complexity of the road network, increased traffic intensity, and the availability of potentially distracting in-vehicle information systems, such as phones, (e.g., Carsten and Brookhuis, 2005), accurate assessment of user state while driving might be used to benefit driving performance. From driving behavior literature, it is clear that besides mental workload, other, related psychological constructs might be investigated for use in a support system. At this point there is no consensus about the exact psychological processes underlying driving behavior. Depending on the theoretical framework, the level of (subjective) risk, workload, or a general feeling of comfort is either maintained or avoided (e.g., risk homeostasis theory, the zero-risk theory, risk allostasis theory, safety margin model (Näätänen and Summala, 1976; Wilde, 1982; Fuller, 2005; Summala, 2005; see also: Lewis-Evans et al., 2011). To make it even more complex, drivers alter the level of workload in practice through behavioral adaptations. For example, in demanding situations with high information density (e.g., complex variable message signs), narrow lanes or a winding road, a driver may reduce speed, which will reduce the reaction time requirements, and thereby avoids high workload levels (Hockey, 2003; Lewis-Evans and Charlton, 2006).

Ultimately, we would like to provide a proof of concept for a passive brain-car interface that changes driving speed in response to visuomotor workload, thereby keeping workload levels within an acceptable range, similar to a human driver. However, in preparation for this, we have first investigated the feasibility of using EEG signals to classify between levels of lane keeping demand in a driving simulator. For this, we applied subject-specific Common Spatial Patterns (CSPs; e.g., Blankertz et al., 2008). The main advantage of using the CSP technique is that it maximizes the difference between two conditions by creating linear combinations of all included electrodes; spatial filters used to produce CSP components. In this way, some electrodes contribute more to the filtered signal(s) than others. These CSP components are determined per participant and therefore, individual differences are accounted for. The most discriminative components are then used to distinguish conditions.

Lane keeping demand was manipulated by changing driving speed, mimicking drivers' natural behavior. Driving speed was set relative to the participants' comfortable speed, since the effort that is required to keep the car safely on the road may vary between drivers for absolute driving speeds. A relative high driving speed is hypothesized to result in a relative high visuomotor workload. In addition, since the Standard Deviation of the car's Lateral Position (SDLP) reflects workload (e.g., Dijksterhuis et al., 2011), an individually set target SDLP was presented to the participants on the virtual windshield, urging drivers to show less swerving behavior in the driving lane. A relative low target SDLP is hypothesized to result in a relative high workload level.

## Materials and methods

### Participants

A total of 17 males and 17 females were recruited through social media and poster announcements throughout the University of Groningen and were paid 20 Euros for participation. A large part of the participants were either Dutch or German students at this university. Ages ranged from 21 to 34 years (*M* = 24.0; *SD* = 3.0) and the participants had held their driver's license for 3 to 15 years (*M* = 5.3; *SD* = 2.8). Self-reported total mileage ranged from 3000 to 350,000 km (*M* = 53,000; *SD* = 76,000), while the reported average annual mileage over the past 3 years ranged from 1000 to 50,000 km (*M* = 9000; *SD* = 11,000). None of the participants reported on using prescribed drugs that might affect driving behavior. The Ethical Committee of the Psychology Department of the University of Groningen has approved the study.

### Simulator and driving environment

The study was conducted using a fixed-base vehicle mock up with functional steering wheel, indicators, and pedals. The simulator runs on ST Software© which is capable of simulating fully interactive traffic. The three computers dedicated to the simulator compute the road environment and traffic which are displayed on three 32-inch plasma screens and provide a total view of the driving environment of 210°. In addition, three rear-view mirrors are projected on the screens. A detailed description of the driving simulator used can be found in Van Winsum and Van Wolffelaar (1993).

For the experiment a two-lane road (each 2.75 m wide) was prepared, without intersections and winding through rural scenery. The road consisted mainly of easy curves (about 80%) with a constant radius of 380 m and ranging in length from 120 to 800 m. The road surface was marked on the edges by a continuous line (0.20 m wide) and in the center by a discontinuous (dashed) line (0.15 m wide). Outside the edges a soft shoulder was present and there were no objects in the direct vicinity of the road. In the driving direction of the participants, no traffic was present. However, oncoming traffic, travelling between 76 and 84 km/h, was generated with a random interval gap between 1 and 2 s, resulting in 40 passing private vehicles per minute on average. The speed of the participant's vehicle was controlled by the simulator for all rides during the experimental session, except for the initial ride during which the participants drove the simulator car (width: 1.60 m) in automatic gear mode.

### Design and procedure

Upon arrival at the experimental site of the University of Groningen, a participant was informed in general terms with respect to the experimental design, was requested to sign an informed consent form, and asked to fill in a short questionnaire mainly related to their driving experience. Hereafter, the participant was given some time (ca. 7 min) to practice driving in the simulator, before the sensors were attached. Next, a three minute baseline recording was made while the participant sat in the simulator car chair and an aquatic movie played on the center screen of the simulator.

After this baseline recording the participant completed 16 short rides. After each ride, the participant was requested to park the vehicle on the side of the road and to provide an answer to two brief questions (on subjective mental effort and estimated driving speed). During the initial ride (140 s) the participant exerted both longitudinal and lateral control over the vehicle and was asked to find and drive at a speed that felt most natural and comfortable in this situation while the speedometer was turned off to prevent rule-based speed setting. The speedometer remained turned off for the entire experiment. The mean speed and standard deviation of the vehicle's lateral position (SDLP) on the road during the last 110 s of the initial ride represented the participant's personal, comfortable driving style. These parameters were saved and used to set driving speed and target SDLP during the 15 remaining rides.

During these 15 rides (130 s each), speed was set relative to the participant's comfortable speed (either −40, −20, 0, +20, or +40 km/h). In addition, while speed was set at the comfortable driving speed, the participant was requested to keep SDLP at either 0, −0.05, or −0.10 m relative to the initial SDLP, which represent a normal, hard, or very hard task. For the other driving speeds, the target SDLP was determined as follows. From a pilot study (*n* = 9), using a similar roadway environment, it was found that SDLP naturally increases as a function of speed. To compensate for this effect and thereby creating five roughly comparable steering challenges across speeds, another 0.03 m per speed level was either added to or subtracted from the target SDLP. For example, when driving 40 km/h slower than the comfortable speed while the target SDLP condition was set at “very hard,” the numerical target SDLP was set 0.10 + 2 × 0.03 = 0.16 m lower than the comfortable SDLP as established during the initial ride. Current values of SDLP were derived from a 15 s moving window which was updated every second and these were projected onto the bottom of the windshield of the simulator while driving, adjacent to the target SDLP. In this way a driver could monitor real SDLP and compare it to the target. Accounting for the time window and for the time the simulator needed to get to the required speed, only the last 110 s of each ride was used in subsequent analyses. To be clear, the data used for this analysis were the raw, not averaged, vehicle parameters. In total, the experimental manipulations resulted in a within-subject design consisting of two repeated measures factors with several levels: speed (5) and target SDLP (3). The participants were exposed to these driving conditions according to a randomized schedule.

After finishing the last ride, the baseline measurement was repeated once more before all physiological sensors were removed. Finally, the participants were debriefed and were paid upon leaving.

### Dealing with collisions

Occasionally, the participants were challenged to the point that a collision with oncoming traffic could not be avoided. In total, six participants were involved in 10 collisions which is 1.8% of all experimental rides. Eight of these collisions occurred in a +40 km/h speed condition. When a collision occurred, that particular ride was repeated. Data acquired during the crash rides were not used for further analyses.

### Data acquisition

#### Vehicle parameters

Driving speed and lateral position (LP) were sampled at 10 Hz. LP is defined as the difference in meters between the center of the participant's car and the middle of the (right hand) driving lane. Positive LP values correspond to deviations toward the right hand shoulder and negative values correspond to deviations toward the left hand shoulder. The sampled LP values were processed while driving and used to calculate mean LP and SDLP for each of the 16 rides. In addition, LP values were used to feed current values of SDLP back to the participant which were calculated by using moving, overlapping time windows (see Design and Procedure for more details), representing an indication of the participants lane keeping performance.

#### Subjective ratings

After each ride, a rating on the one-dimensional Rating Scale Mental Effort (RSME) was requested (Zijlstra, 1993). The RSME ranges from 0 to 150 and several effort indications are visible alongside the scale which may guide the participant in marking the scale. Indications start with “absolutely no effort” (RSME score of 2) and end with “extreme effort” (RSME score of 112). The participants, who did not receive speed information from the speedometer, were also asked to write down an estimate of the driving speed they just experienced.

#### Physiological measures

Physiological signals were sampled at 250 Hz. Firstly, the electrocardiogram (ECG) was registered using three Ag-AgCl electrodes, which were placed on the sternum (the ground electrode) and on the right and left side between the lower ribs. However, given the emphasis on brain activity in this paper, the ECG results are not reported here. Secondly, the electro-oculogram (EOG) was measured by Ag-AgCL electrodes attached next to the lateral canthus of each eye and above and below either the right or left eye. The EEG was measured using an electro-cap with 64 tin electrodes (at the following sites: FP1, FP2, Afz, F7, F5, Fz, F4, F8, T7, C5, C3, Cz, C4, T8, P7, P3, Pz, P4, P8, O1, and O2.) The amplifer was a REFA 8–72 (Twente Medical Systems International, Enschede, The Netherlands). Portilab 2 software was used to record all physiological signals. The ground electrode used for the ECG recording also served as the participant's ground for the EEG recording. EEG and EOG signals were amplified with a 1 s time constant (0.016 Hz high-pass). All EEG channels were referenced against the average activity of all channels during the registrations. In addition, a reference electrode was attached to each mastoid. Impedances were kept below 10 kΩ for all electrodes.

### EEG data processing

Starting from the raw EEG signals, the sampled EEG and EOG data were first high-pass filtered (cutoff = 0.3 Hz, at 12 dB/Oct Butterworth filter) before the EEG data segments of the 15 experimental conditions (110 s each) were corrected for eye movements and blinks, using Brain Vision Analyzer (Gratton et al., 1983). The corrected data segments were then exported into binary files. No data epochs were removed before further processing.

The exported data files were processed using MATLAB R2010a (The MathWorks, Inc., USA, www.mathworks.com). After importing two data sets (two rides or conditions) of a particular participant, the EEG was band-passed filtered in the frequency domain (FFT filter) of interest, using an edge frequency of 1 Hz below and above the lower and upper frequency band limit respectively. The imported data (110 s for each condition) were then segmented into one second epochs and baselined relative to each mean activity. The first and last 10% of the epochs were omitted, leaving the 88 middle, non-overlapping, epochs per condition in the cross-validation design. This entailed a repeated (50 times) random portioning of two data classes (a condition pair) into a set of 66 training epochs (75%) and a set of 22 test epochs for each data class. The training sets were used to train the participant-specific classifier that was subsequently used to classify the testing epochs of each data class. This iteration process was carried out for each included participant, frequency band, EEG cap configuration, and data pair. The accuracies reported in the result section reflect the average accuracies across all 50 iterations and all included participants.

To improve discriminatory power of the data classifier, the contrast between two data classes was optimized by using the CSP technique. This technique determines CSP filters in such a way that they maximize the variances of spatially filtered signals for one training set while minimizing them for the other (Blankertz et al., 2008). A CSP filter is a coefficient vector by which the original channels can be transformed. This results in a new spatially filtered channel (a CSP component) which is a linear combination of all original channels, and the total number of filters and therefore, the number components, is equal to the number of original channels. The matrix of CSP filters is determined by solving a generalized eigen-value problem. The filter corresponding to the largest eigen-value yields a high variance signal in one condition, while producing a low variance signal in the other; and vice versa for the filters corresponding to the smallest eigen-value. The CSP filters are therefore ranked according to these eigen-values and the first and last filters in this sorted W matrix are usually used for further classification. To be more specific, in the current study, the two, four, or six filters (always an equal number from each side of the sorted W matrix) that resulted in the largest difference in variance between two training sets was used. Next, the total variance per training epoch and per CSP component was calculated and their logarithms were taken before entered into Fisher's linear discriminant analysis. This analysis again transforms the data by determining the linear weights of the discriminant function that combines data points of the two training sets in such a way that maximizes the distance between them. Finally, the CSP filters and classifier weights were used to classify the remaining testing epochs of the two conditions.

A wide range of EEG frequency bands were explored to investigate where useful discriminatory information might be present. Four frequency search strategies were deployed. The first frequency search strategy was characterized by both an increasing high pass cut-off point (increasing 1 Hz for each iteration) and an increasing frequency bandwidth (1.5 times the low frequency band limit). At the first iteration, frequencies between 3 and 4.5 Hz were passed. At the last iteration, frequencies between 72 and 108 Hz were passed. The second strategy entailed exploring all frequencies between 3 and 70 Hz using a fixed bandwidth of 1 Hz. For the third strategy, bandwidth was set to 4 Hz and iterations ran from 4 to 72 Hz. Lastly, data was filtered in broad bands to classify between conditions; 8–30, 32–54, 56–78, and 80–102 Hz.

In addition, several EEG cap configurations were explored. To start with, all 21 EEG channels were included. To explore whether classification accuracy may differ between scalp regions, several subsets were defined and tested. Firstly, a peripheral set was defined, consisting of 14 electrodes, (FP1, FP2, Afz, F7, F5, F4, F8, T7, C5, T8, P7, P8, O1, and O2). Secondly, a frontal set consisting of 7 electrodes (FP1, FP2, F7, F5, Fz, F4, and F8), which are associated with executive functions that are important in driving. Thirdly, a posterior set consisting of 7 electrodes (P7, P5, Pz, P4, P8, O1, and O2), containing electrodes associated to visuomotor processing. Lastly, the electrode set identified by Prinzel III et al. (2001) (P3, Pz, P4, Cz), which has often been used in adaptive automation research to get the “engagement index” [defined as the ratio; beta/(alpha + theta)].

Lastly, five condition pairs were selected from a total of 105 possible combinations (15!/2!(15–2)!). An experimental condition can be defined in terms of its driving speed level and target SDLP difficulty level. To improve comparability one factor was kept constant for each condition pair. In this way, four speed differences for the normal target level were classified: −40 vs. +40 km/h, −20 vs. +20 km/h, −20 vs. 0 km/h, and 0 vs. +20 km/h. The normal target level was chosen since this target resembles the individuals' natural driving behavior. Focusing on classifying between speed differences in this way was done because of the envisioned application. A brain-based adaptive cruise control would change speeds and therefore, the effect of speed interventions has to be assessed. In addition, as it turned out, the very hard target conditions required more subjective effort compared to the normal target level, and therefore, these two conditions were compared in the 0 km/h relative speed condition.

Due to data anomalies such as missing channels, eight participants were excluded from the offline classification phase of this study. Despite a smaller participant pool, the number of condition pair comparisons is very large: 161 frequency bands × 5 condition pairs × 5 EEG cap configurations × 26 participants × 3 numbers of components = 313,950. Given these large numbers, only a selection of aggregated classification accuracy values can be reported (Figures 2, 3) next to examples of scalp topographies of CSP components (see Figure 4 for an impression) reflecting how the information sources project to the scalp (retrieved from the inverse of W; see Blankertz et al., 2008).

## Results

### Vehicle parameters and subjective ratings

**Figure 1:**
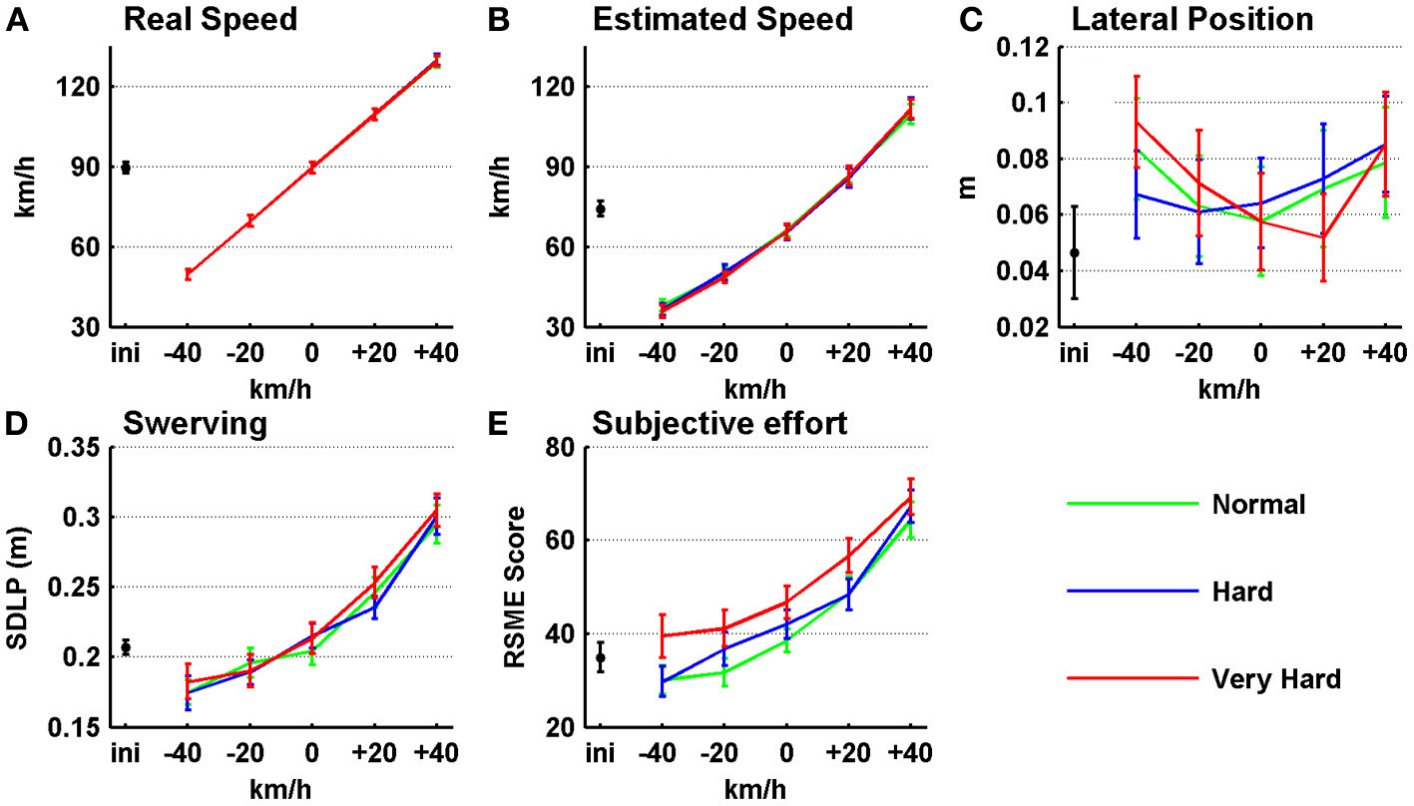
**Vehicle parameters and subjective ratings as a function of set driving speed condition. (A)** Real driving speed. **(B)** Estimated driving speed. **(C)** Lateral Position (LP). **(D)** Standard Deviation of the Lateral Position (SDLP). **(E)** Rating Scale Mental Effort (RSME). On the x-axes, values for the initial ride (black dots) are shown in addition to five driving speeds that were set, relative to the individual's preferred driving speed established during the initial ride. Error bars represent the standard error. LP values represent the middle of the car (car width = 1.60 m) in relation to the middle of the right (driving) lane (width = 2.75 m). Normal, hard, and very hard indicate the difficulty of keeping current SDLP values under the target SDLP: see section Design and Procedure for details. Positive LP values indicate a position to the right hand of the lane mid. Maximum score for mental effort is 150. *n* = 34.

Subjective ratings and vehicle parameters are shown in Figure 1 and their test outcomes are listed in Table 1. To begin with, the participants' preferred speed during the initial ride ranged between 62 and 120 km/h, averaging at 90 km/h (see the black dot in Figure 1A). This is slightly faster than estimated for this ride (*M* = 74 km/h; Figure 1B). This pattern of underestimating driving speed is present for all speed levels (Pearson's product moment correlation =0.99 over all conditions).

**Table 1:** **Multivariate test results for vehicle parameters and subjective effort ratings (Figure 1)**.

**Vehicle parameters and subjective ratings**									
***Effect***	**LP**			**SDLP**			**RSME score**		
	***F*_(df1,df2)_**	***p***	**η^2^_*p*_**	***F*_(df1,df2)_**	***p***	**η^2^_*p*_**	***F*_(df1,df2)_**	***p***	**η^2^_*p*_**
Speed (S)	**4.46 (4,30)**	**0.006**	**0.373**	**45.40 (4,30)**	**<0.001**	**0.858**	**21.10 (4,30)**	**<0.001**	**0.748**
Target (T)	0.26 (2,32)	0.974	0.002	1.32 (2,32)	0.283	0.076	**08.49 (2,32)**	**0.001**	**0.347**
S ×T	0.77 (8,26)	0.633	0.191	1.22 (8,26)	0.324	0.274	1.25 (8,26)	0.309	0.278

LP, Lateral Position; SDLP, Standard Deviation Lateral Position; RSME, Rating Scale Mental Effort. Significant effects (p < 0.05) are shown in bold. Speed effect relates to speed condition, Target to SDLP target.

The dimensions of the vehicle and driving lane allowed for 0.58 m of swerving margin on both sides of the vehicle. As can be seen in Figure 1C, the participants stayed well within their driving lane on average and positioned the vehicle slightly toward the right hand shoulder (0.07 m on average). As can be read in Table 1, there was a significant effect of speed on LP. The participants' mean position on the road curves toward the right-hand shoulder, both when driving slower and faster than the preferred speed (polynomial contrasts showed a quadratic trend; [*F*_(1, 33)_ = 15.35, *p* < 0.001, η^2^_*p*_ = 0.317]. Next, as speed increased, so did the participants' mean SDLP (see Figure 1D), representing swerving behavior, from 0.18 m during the slowest speed to 0.30 m during the fastest speed. This is mainly a linear increase [*F*_(1, 33)_ = 182.81, *p* < 0.001, η^2^_*p*_ = 0.847], although SDLP increases slightly more rapidly toward the higher speeds [quadratic trend; *F*_(1,33)_ = 24.33, *p* < 0.001, η^2^_*p*_ = 0.424]. Note that the factor; target SDLP, indicating the difficulty of keeping current SDLP values under the target SDLP while driving, had no effect on the actual SDLP. In addition, interactions between speed and target SDLP are not present in the data.

Figure 1E shows that the mental effort ratings increased from between “a little effort” and “some effort” (a mean RSME score of 33) for the slowest speeds to between “rather much effort” and “considerable effort” (a mean RSME score of 34) for the fastest speeds [linear trend; *F*_(1, 33)_ = 88.48, *p* < 0.001, η^2^_*p*_ = 0.728]. Also, similar to SDLP, this increase is stronger toward the faster speeds [quadratic trend; *F*_(1, 33)_ = 86.04, *p* < 0.001, η^2^_*p*_ = 0.327]. In addition, even though target SDLP did not have an effect on vehicle parameters, there was a main effect on mental effort ratings. Bonferroni corrected pairwise comparisons revealed that the “very hard” level was perceived as more difficult than the other two, while “normal” and “hard” did not show a difference.

### Classification results

#### Averages classification accuracies

**Figure 2:**
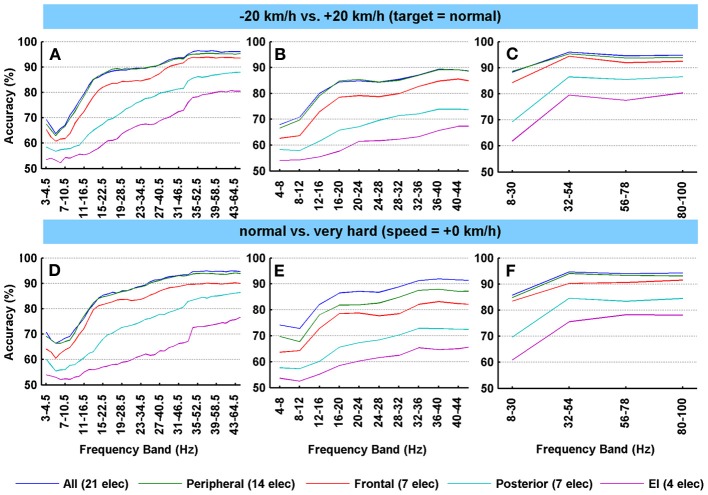
**Average classification accuracies of the Fisher's linear discriminant analyses after spatial filtering for several condition pairs. (A–F)** The accuracy values represent the average subject-specific classification accuracy over all participants that resulted from the cross-validation scheme. Classifications were based on applying the two most contrasting CSP components to the EEG channels. *N* = 26. For each row of subfigures, a different EEG cap configuration was used. For the left column **(A,D)**, the frequency bandwidth is 1.5 times the start frequency (step size 1 Hz), starting at 3–4.5 Hz and ending at 43–64.5 Hz. For the middle column **(B,E)**, 4 Hz bands were used and a stepsize of 4. For the right column **(C,F)**, a broad band frequency search (22 Hz) was deployed. All electrodes: FP1, FP2, Afz, F7, F5, Fz, F4, F8, T7, C5, C3, Cz, C4, T8, P7, P3, Pz, P4, P8, O1, O2. Peripheral set: FP1, FP2, Afz, F7, F5, F4, F8, T7, C5, T8, P7, P8, O1, O2. Frontal set: FP1, FP2, F7, F5, Fz, F4, F8. Posterior set: P7, P5, Pz, P4, P8, O1, O2. Engagement index (EI) set: P3, Pz, P4, Cz.

**Figure 3:**
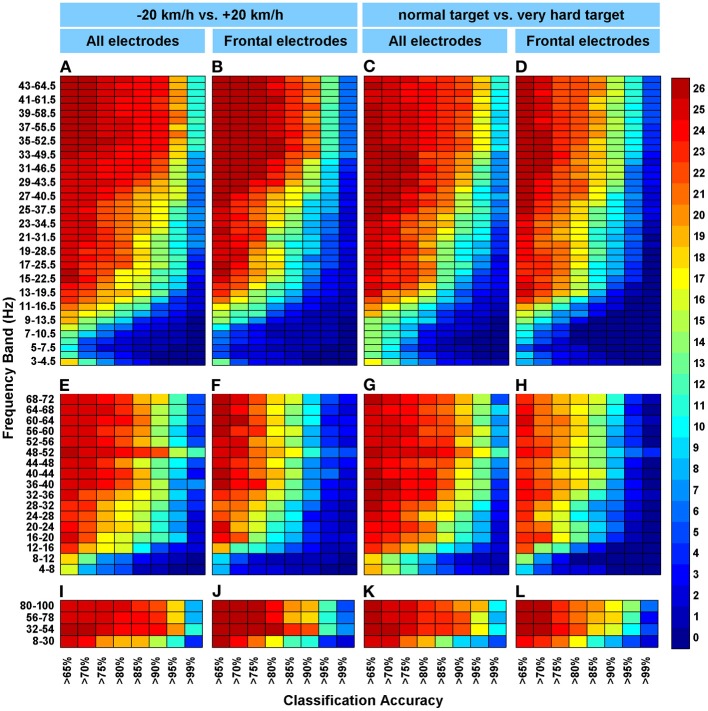
**The cumulative frequencies of classification accuracies. (A–L)** Colors represent the number of participants for whom a particular accuracy was found or better (max = 26 participants) in the accuracy category displayed on the x-axes. Subfigure columns are arranged by EEG cap configuration (all electrodes or the frontal set) and by classified condition pair. All electrodes: FP1, FP2, Afz, F7, F5, Fz, F4, F8, T7, C5, C3, Cz, C4, T8, P7, P3, Pz, P4, P8, O1, and O2. Frontal electrodes: FP1, FP2, F7, F5, Fz, F4, and F8. “−20 km/h vs. +20 km/h” indicate set driving speeds relative to the participants' preferred speed as determined during the initial ride (at normal target level). “Normal target vs. hard target” indicate performance target difficulty (at relative speed = 0 km/h). For each row of subfigures, a different frequency search strategy was used. **(A–D)** For the top row of subfigures, the frequency bandwidth is 1.5 times the begin frequency (step size 1 Hz), starting at 3–4.5 Hz and ending at 44–66 Hz. **(E–H)** For the middle row of subfigures, 4 Hz bands were used and a step size of 4. **(I–L)** For the bottom row, a broad band frequency (22 Hz) search was deployed.

In Figure 2, the classification accuracies for several condition pairs are shown. Figure 2 (and Figure 3) only shows the average classification accuracies for two data pairs (−20 km/h vs. +20 km/h and normal performance target vs. very hard performance target). Although more extreme driving speed conditions could have been shown (e.g., 40 vs. +40 km/h), we feel that more similar speed conditions better reflect real driving circumstances and are therefore more relevant. Also, accuracy levels across condition pairs tended to be similar, and therefore the number of shown condition pairs was limited.

**Figure 4:**
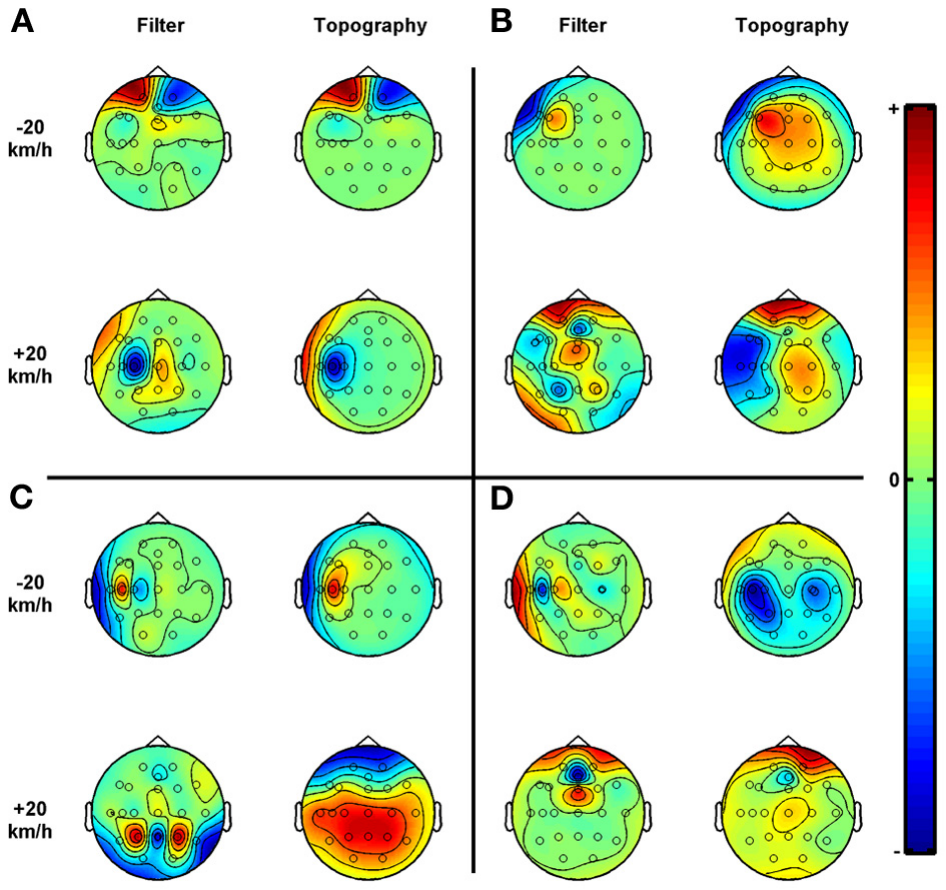
**Examples of CSP analyses. (A–D)** The scalp topography of the components illustrate how the physiological sources project to the scalp. The components are determined such that projected signals are optimally discriminative. The filters and topographies correspond to the first and last vector of the sorted W matrix and its inverse respectively (see section Design and Procedure for more details). Absolute coloring is arbitrary, however, dense red or blue areas show where the greatest differences in the projected signals' amplitudes were found, between the −20 km/h and the +20 km/h set driving speed (at normal target level). These driving speeds were set relative to the participants' preferred speed as determined during the initial ride. Included electrodes: FP1, FP2, Afz, F7, F5, Fz, F4, F8, T7, C5, C3, Cz, C4, T8, P7, P3, Pz, P4, P8, O1, and O2. **(A)** Subject = 13, frequency band = 72–108 Hz, classification accuracy = 100%. **(B)** Subject = 27, frequency band = 8–12 Hz, classification accuracy = 98%. **(C)** Subject = 21, frequency band = 24–28 Hz, classification accuracy = 82%. **(D)** Subject = 25, frequency band = 8–30 Hz, classification accuracy = 74%. Please note that in the CSP literature, a scalp topography of a component is usually referred to as a spatial pattern.

The graphs in Figure 2 reveal several general trends. Firstly, accuracy tends to increase as frequency increases. This can be seen across electrode sets and condition pairs with accuracies reaching levels of 95% on average over all participants when a relative high number of electrodes is included (21 and 14). This increase is most pronounced in the frequencies from 5 to 20 Hz, after which it continues to rise more gradually indicating a ceiling effect (see for example Figure 2A). This ceiling is about 5–10% lower for the middle column of subplots in Figure 2 (displaying the 4 Hz search strategy). Secondly, a broader frequency band tends to yield higher accuracies, which is most apparent when comparing the middle column (Figures 2B,E; 4 Hz frequency bands) to the right column (Figures 2C, 3F; 22 Hz frequency bands). For example, when including all electrodes, the 4 Hz frequency bands in the 8–32 Hz range in Figure 2B range produced about 15% less accuracy when compared to the first broad band (8–30 Hz) in Figure 2C. Thirdly, there are distinct differences in accuracies as a result of using different channel sets. For example, the larger electrode sets (21 and 14 electrodes) yielded very comparable high accuracies, while the smallest (4 electrodes) consistently resulted in lower classification accuracies (about 15–25% less, depending on frequency band). Such differences can be understood in part from the fact that more channels provide a richer, higher-dimension database for the CSP technique to extract useful discriminatory power. Note however, that the seven frontal electrodes outperformed the seven posterior electrodes by about 5–15%, again depending on frequency band. The shape of the frontal curve in all subfigures (the red lines) reflect the upper two curves (all electrodes and 14 peripheral electrodes), while the posterior curves resemble the bottom EI curves. Finally, when focusing on the somewhat lower EEG frequency of Figures 2A,D ranges (e.g., 10–21 Hz), which are more likely to reflect neuronal activity, the mean accuracy in that range over both subfigures is 80% for the larger two electrode sets. The frontal set led to a classification of 76% on average, while the posterior and the engagement index set resulted in 62 and 55% respectively.

#### Cumulative classification accuracies

In Figure 3, cumulative classification accuracies are shown for a selection of classification results. This figure indicates the consistency of classification accuracies across all 26 included participants. For instance, Figure 3A shows that in the high frequency range (e.g., 43–64.5 Hz), test data from 10 participants were accurately classified 99% of the time or better. Figure 4 confirms that higher frequencies usually yield better accuracies as the top frequencies in all subfigures display more red/yellow than the bottom frequencies. The green/yellow colors indicate that about half to two third of the participants were above the classification threshold indicated on the x-axes. When viewing these colors in Figure 3 through the eyelashes, it can be seen that, especially for the larger electrode set (Figures 3A,E,I and 3C,G,K), data from a substantial number of participants still yielded 85% + accuracy in the lower (alpha and beta) frequency ranges (e.g., 10–20/30 Hz). For instance, the classifier could accurately classify (85% or better) between −20 and +20 km/h in the 16–20 Hz frequency range for 16 out of 26 participants (Figure 3G). For the smaller, frontal electrode set (Figures 3B,F,J and 3D,H,L) the number of participants yielding highly accurate classifications is somewhat less in the lower frequency range; as indicated by the larger presence of blue colors.

#### Example common spatial pattern analysis

Figure 4 displays several CSP filter-topography pairs which are meant to illustrate the diversity of CSP scalp topographies. A common topography across participants, reflecting how the neurological sources project to the scalp, was not identified. However, we selected these topographies based on their resulting classification accuracies and/or the fact that the frequencies are within the normal EEG range. To start with, Figure 4A shows that for participant 13, the perfect classification accuracy in the broad 72–108 frequency range originates mainly from the frontal electrodes (Fp1 and Fp2) which were highly specific for the -20 km/h driving condition, and from C5 which was highly specific for the +20 km/h driving condition. This is illustrative for the general finding that the frontal electrodes were often the main contributors to very high classification accuracies. The other subfigures show topographies linked to frequencies below 30 Hz. In Figure 4B, topographies are shown that resulted in an unusually accurate classification for this relative low frequency band (98% in the 8–12 Hz, alpha, frequency band). In this case, the topographies are more distributed over the scalp, although the left temporal and frontal regions were important physiological sources for discriminating between the two data classes. The scalp topography for the +20 km/h condition in Figure 4C shows a central-parietal distribution, illustrating that the EI electrodes: P3, Pz, and P5 contributed to the 82% classification accuracy in the high beta range of participant 21. The −20 km/h topography suggests that C5 was by far the most distinctive electrode when maximizing the variance of the projected signals in this data class while minimizing it for the other. Finally, Figure 4D shows the CSP resulting in 74% classification accuracy for subject 25 in the broad 8–30 (alpha plus beta) Hz frequency band. These topographies suggest that discriminative power was distributed over the posterior electrodes in the −20 km/h condition and more evenly distributed over the scalp in the +20 km/h condition.

## Discussion

The aim of the study was to investigate the feasibility of using EEG for monitoring the level of visuomotor workload in a driving environment, which can potentially be used by an user adaptive driver support system. To manipulate workload, we exposed drivers to five levels of driving speed that were set relative to their preferred driving speed. In addition, since increasing steering effort normally decreases swerving behavior within the driving lane given a particular speed, participants were presented with three explicit swerving performance targets represented as the standard deviation of the lateral position of the car with respect to the driving lane. To distinguish between workload levels, subject-specific CSP and linear discriminant analysis based classification models were used.

To begin with, subjective mental effort data show that driving at a higher speed is indeed experienced as requiring more effort. Furthermore, estimated driving speed was slightly lower than the real driving speed. Previous research has shown that driving speed in a simulator, when driving on straight roads or easy curves, tends to be higher than it would be on real roads (e.g., Bella, 2008). This effect could be caused by a difference in speed perception between the real world and (fixed-base) driving simulators due to the absence of several speed cues, such as car movements and stereoscopic depth perception. During the current study these factors may also have contributed to misjudging driving speed, especially since the speedometer was hidden from view at all times. The standard deviation of the lateral position (SDLP), indicating lane keeping performance, increased as function of speed, which is normal (e.g., Peng et al., 2013). However, the performance target (target SDLP) did not have an effect on vehicle parameters, suggesting that this manipulation failed since a decrease of SDLP was expected if participants were exposed to more difficult target SDLPs. However, participants did rate the “very hard” target SDLP condition as the most difficult, perhaps demonstrating that participants were trying hard but could not manage. Also, EEG data from the very hard SDLP condition could be accurately discriminated from data acquired during the normal SDLP condition which is another indication that participants did not simply ignore the instructions. Since other task manipulations aimed at increasing steering difficulty, such as decreasing lane width, have proven to affect SDLP (e.g., Dijksterhuis et al., 2011), the absence of an effect on SDLP may be explained by this particular manipulation. In contrast to the automatic nature of the steering task during normal driving participants had to actively engage themselves in transferring numerical information about their lane keeping behavior, as presented on their windshield, to steering wheel movements.

EEG activities during the experimental conditions were classified, yielding several interesting results. Firstly, applying CSP to a variety of frequencies and frequency band widths revealed that, overall, broader bands and higher frequencies result in higher classification accuracies. This could be taken to suggest that neuronal gamma synchronization correlated with the task manipulations in which case these results are in line with other research suggesting that activity in the gamma frequencies reflects sensory-motor coordination (Schoffelen et al., 2005; see also Fries et al., 2007). However, this conclusion should be drawn with caution since muscle activity as represented in the EMG has power in the same frequencies, which is picked up by EEG electrodes as well (Whitham et al., 2007; Muthukumaraswamy and Singh, 2013). This view of muscular activities contributing to high classification accuracies in the gamma band is confirmed by graphs showing the projections of the CSP components. Figure 4A demonstrates just one case where the perfect classification for high frequencies can mostly be traced to the EEG electrodes close to the eyes. However, a relative high contribution of the peripheral electrodes for extremely high classification accuracies is an emerging pattern. Moreover, when performing a semi-real task, such as driving in a simulator, EMG activity can be expected to be more dominantly present compared to more controlled laboratory tasks, making classifications based on neuronal gamma activities less likely.

High accuracies were also found for a substantial number of participants in the lower frequency ranges, as shown in Figures 2 and 3, and these are unlikely to be confounded by EMG activity. As shown by Whitham et al. (2007), who recorded EEG during paralysis by neuromuscular blockade, EMG activity is largely absent from frequencies below 20 Hz. Therefore, we suggest that classifications in the lower frequency ranges were likely determined by underlying neuronal activity.

CSP component topographies showed no readily discernible degree of consistency across participants, as illustrated in Figure 4. This indicates that the effects of changes in psychological construct such as mental workload on electrical activities on the scalp is very subject dependent, which confirms that individually tuned classification approaches are required for accurate classifications. In case of high frequencies this implies that the, perhaps subconsciously produced muscular activities, show large inter-individual variations. In case of the lower frequencies, it is likely that also on a neurological level, there are large variations. Finding consistent topographies would have been promising for future applications. For example, it could lead to a theory-driven pre-selection of scalp locations, thereby excluding possible irrelevant information from the classification model. Yet, it may be expected to find a large inter-individual variability when classifying rather abstract mental states compared to, for example, classifying the difference between left and right hand motor imagery for which the neuroanatomical base is much clearer.

A limitation of the current study is that the experimental conditions (rides) could not be randomized within each participant. e.g., changing speed conditions every couple of seconds would have resulted in a highly unnatural driving experience. The drawback of the used approach is that there was an average of about 15 min between one condition and the other within each condition pair that was used for the classifications. Since neighboring epochs can be similar to each other, a difference in time may have led to an inflation of the classification accuracies. For future research, it is advised to repeat conditions within subjects to assess the potential effects of time dependencies. For example, by training the classifier on one condition pair and validating it on the other, identical condition pair. While it is important to realize that time dependencies cannot be ruled out, it should also be noted that it probably did not affect other effects, such as the accuracy difference between the parietal and frontal electrode set or the difference between low and high EEG frequencies.

Overall, these findings imply that the subject-specific CSP approach provides very good discriminatory power between visuomotor workload conditions over a large range of frequency bands. With respect to the high (gamma) frequency ranges it is important to realize that major contributions from muscular activities cannot be ruled out. Moreover, this will probably be true for most passive BCI applications as real life tasks, such as driving a car, usually require a lot of motor activity. A workload classification strategy based on EMG activity would therefore be worthwhile investigating in future research, which requires a relative low number of electrodes. However, high classification accuracies were also found for the lower EEG frequencies, implying a large contribution of neurological activities. These high accuracies are promising for future applications, however, several issues need to be addressed before a system is working from the user's point of view. Some of these issues will be further discussed below.

Even if classification accuracies of up to 80% may be considered quite high for 1-s epochs, it raises the issue of applicability; especially when performing a safety-critical task, this seems insufficient. However, depending on the temporal responsiveness requirements of an application, these accuracy levels might suffice. For example, using longer data epochs can be expected to result in more accurate classifications, since more information is available to the classifier (e.g., Brouwer et al., 2012). Although not further reported in the result section, increasing the epoch length from 1 to 2 s was found to increase accuracies with about 3% for the lower frequency ranges. Another option would be to combine several successive small data epochs. As an illustration, assuming that successive classifications are independent and applying a simple binomial chance distribution, then combining five successive epochs, each having a 80% chance of accurately being classified, would lead to a 94% accuracy when using a majority vote (i.e., three or more epochs are classified correctly). This would decrease the negative effects of small periods of noisy data which may be expected in real life tasks and which should improve a system's behavior from the users point of view.

Another important issue that needs to be solved before reliable applications can be build are the so-called non-stationarities in EEG signals, which refer to shifts in EEG signals between the initial calibration session during which a model is trained and online application. Non-stationarities negatively impact the transfer of classification accuracies between calibration and application of a model (e.g., Shenoy et al., 2006). One solution to this issue could be to update the classification model from time to time by adding additional calibration periods when the task at hand allows for it. Another solution are adaptive classifiers, which use data that are acquired while the user is interacting with the system in real-time (Shenoy et al., 2006). The drawback of using an adaptive classifier is that it requires immediate labeling of new, incoming data while the user is engaged in task performance. In some active BCI systems, for example, when controlling a game, it is plausible that the required information is available. In case of a passive BCI system however, this is most likely not the case. Again, using longer periods of time may offer a solution to this problem. For example, assuming that mental workload does not vary every second, all EEG data measured over a somewhat longer period reflect one particular level of workload. If the classifier therefore classifies most epochs as data class A, then all epochs in that period could be labeled as such and subsequently used to update the classification model. Finding acceptable and robust methods of updating the classification model is likely to be a necessary development before (passive) BCI systems can be applied to task situations.

For the viability of future applications it is also important that the binary approach of discriminating between two data classes is expanded to the multiclass situation. For instance, workload levels during task performance may be either too high, too low or within an acceptable range. In an adaptive system, where support may be changed, activated, or deactivated based on workload classifications it is therefore of equal importance that the conditions for no change are defined. Thus, in terms a passive BCI application, a homeostatic system aimed at keeping workload at or around optimal levels, must also “know” when not to initiate changes. One way to accomplish multi-class analyses is to combine several pairwise classifications through voting procedures (Friedman, 1996; see also Dornhege et al., 2004; Grosse-Wentrup and Buss, 2008).

In conclusion, depending on temporal responsiveness requirements, a system's designer may have the option to either focus on high EEG frequencies and accept that muscular activities likely contribute to classification accuracies, or to focus on lower EEG frequencies that mainly reflect neurological activities but accept slightly lower accuracies. Although it is clear that the very high classification accuracies found in this offline study by themselves do not guarantee a well-functioning online system, it is a promising start in realizing a CSP based passive BCI system that can reliably be used to monitor visuomotor load in real-time.

### Conflict of interest statement

The authors declare that the research was conducted in the absence of any commercial or financial relationships that could be construed as a potential conflict of interest.
